# A key role for an impaired detoxification mechanism in the etiology and severity of autism spectrum disorders

**DOI:** 10.1186/1744-9081-10-14

**Published:** 2014-04-28

**Authors:** Altaf Alabdali, Laila Al-Ayadhi, Afaf El-Ansary

**Affiliations:** 1 Biochemistry Department, Science College, King Saud University, P.O box 22452, Zip code 11495 Riyadh, Saudi Arabia; 2 Autism Research and Treatment Center, Riyadh, Saudi Arabia; 3 Shaik AL-Amodi Autism Research Chair, King Saud University, Riyadh, Saudi Arabia; 4 Department of Physiology, Faculty of Medicine, King Saud University, Riyadh, Saudi Arabia; 5 Medicinal Chemistry Department, National Research Centre, Dokki, Cairo, Egypt

**Keywords:** Autism Spectrum Disorder, Social Responsiveness Scale (SRS), Childhood Autism Rating Scale (CARS), Lead, Mercury, Glutathione-s-transferase, Vitamin E

## Abstract

**Background:**

Autism Spectrum Disorders (ASD) is a syndrome with a number of etiologies and different mechanisms that lead to abnormal development. The identification of autism biomarkers in patients with different degrees of clinical presentation (i.e., mild, moderate and severe) will give greater insight into the pathogenesis of this disease and will enable effective early diagnostic strategies and treatments for this disorder.

**Methods:**

In this study, the concentration of two toxic heavy metals, lead (Pb) and mercury (Hg), were measured in red blood cells, while glutathione-s-transferase (GST) and vitamin E, as enzymatic and non-enzymatic antioxidants, respectively, were measured in the plasma of subgroups of autistic patients with different Social Responsiveness Scale (SRS) and Childhood Autism Rating Scale (CARS) scores. The results were compared to age- and gender-matched healthy controls.

**Results:**

The obtained data showed that the patients with autism spectrum disorder had significantly higher Pb and Hg levels and lower GST activity and vitamin E concentrations compared with the controls. The levels of heavy metals (Hg and Pb), GST and vitamin E were correlated with the severity of the social and cognitive impairment measures (SRS and CARS). Receiver Operating Characteristics (ROC) analysis and predictiveness curves indicated that the four parameters show satisfactory sensitivity, very high specificity and excellent predictiveness. Multiple regression analyses confirmed that higher levels of Hg and Pb, together with lower levels of GST and vitamin E, can be used to predict social and cognitive impairment in patients with autism spectrum disorders.

**Conclusion:**

This study confirms earlier studies that implicate toxic metal accumulation as a consequence of impaired detoxification in autism and provides insight into the etiological mechanism of autism.

## Introduction

Autism is characterized by a set of repetitious behavior in combination with social, cognitive and communication deficits [1]. An emerging hypothesis states that autism may result from a combination of genetic susceptibility and exposure to environmental toxins at critical periods during brain development [2]. Neurotoxins and associated inflammation of the brain tissue are often the focus of therapies for patients with autism. However, if body detoxification is impaired, neuroinflammation will not efficiently improve unless the overall body issues are addressed. Xenobiotics are neurotoxins of external origin, such as chemicals and pollutants in the air, water, food additives and drugs, that can dramatically alter the health of the child. An efficient three-phase mechanism is involved in detoxifying these toxins [3]; however, several factors play a role in modulating this mechanism. For example, age, gender and diet are among the various biological and non-biological factors that modulate individual susceptibility. Additionally, genetic variability plays a critical role in individual susceptibility because variable detoxifier phenotypes result from mutations in the same gene. These range from individuals with regular enzyme and detoxification functions to poor metabolizers with low or no enzyme activity [4,5].

Glutathione-S-transferase (GST) functions in the detoxification of xenobiotics, drugs, toxins, and metabolites and is also involved in the regulation of mitogen-activated protein kinases, which are important during differentiation and development. Hg and Pb, however, are two well-known toxicants that have toxic effects on the body, with the brain being the most susceptible target organ. Exposure to Hg and Pb during pregnancy and early childhood can cause neurodevelopmental impairment and subclinical brain dysfunction because both heavy metals can cross the placenta and the blood–brain-barrier [6]. In the brain, these metals can affect critical developmental processes, including cell proliferation, migration, differentiation, synaptogenesis, myelination, and apoptosis [6].

It is well documented that patients with ASD have many statistically significant differences in their nutritional and metabolic status compared with those who do not have ASD. Some of these biomarkers are indicative of vitamin E insufficiency, increased oxidative stress, and poor detoxification and are associated with the severity of the disorder [7-10]. A recent study found that impaired xenobiotic detoxification is correlated with increased gut permeability (leaky gut) and neuroinflammation, two accepted pathological phenomena in ASD [3].

These findings prompted us to search for biochemical correlations related to the detoxification mechanism and neurobiological processes and the severity and social functioning of patients with ASD measured by the CARS and SRS. We selected Hg and Pb as two environmental toxicants involved in the etiology of ASD, together with GST and vitamin E as enzymatic and non-enzymatic antioxidants with high activity in terminating lipid peroxidation and environmental toxicity. We hypothesized that confirming the relationship between impaired detoxification mechanism and severity of ASD could enhance efforts at early prevention, diagnosis, and intervention and, thus, may play a role in decreasing the prevalence of autism.

## Material and methods

### Subjects

This cross-sectional study was conducted on 52 autistic male children who were recruited from the Autism Research and Treatment Center, Faculty of Medicine, King Saud University, Riyadh, Saudi Arabia. Of these children, 40 were nonverbal, and 12 were verbal. Their ages ranged between 3 and 12 years (mean SD = 7.0 ± 2.34 years). The control group was comprised of 30 age- and sex-matched apparently healthy children with a mean age of 7.2 ± 2.14 years. The patients fulfilled the diagnostic criteria of autism described in the 4th edition of the Diagnostic and Statistical Manual of Mental Disorders. The controls were normally developing, healthy children who were unrelated to the autistic subjects and did not have any of the exclusion criteria. The control children were the healthy older siblings of healthy infants who were attending the Well Baby Clinic at King Khalid University Hospital for routine check-ups of their growth parameters. They had no clinical indications of infectious diseases or neuropsychiatric disorders. All participants had normal results for urine analysis and sedimentation rate. The local Ethical Committee of the Faculty of Medicine, King Saud University, Riyadh, Saudi Arabia, approved this study. In addition, an informed written consent of participation for this study was signed by the parents or the legal guardians of the investigated subjects, according to the Helsinki principles. The selected sample of participants was based on how convenient and readily available the group of participants was (i.e., convenience sampling). Participants were excluded from the study if they had a diagnosis of fragile X syndrome, epileptic seizures, obsessive–compulsive disorder, affective disorders, or any additional psychiatric or neurological diagnoses.

#### *Measurement of Autism severity scales (CARS and SRS)*

The Childhood Autism Rating Scale (CARS) score, which is a measurement of the severity of the disease, rates the child on a scale from one to four in each of fifteen areas (relating to people’s emotional response; imitation; body use; object use; listening response; fear or nervousness; verbal communication; non-verbal communication; activity level; level and reliability of intellectual response; adaptation to change; visual response; taste, smell and touch response and general impressions). According to the scale, children who scored 30–36 had mild to moderate autism (n = 23), while those with scores ranging between 37 and 60 points had severe autism (n = 27) [11,12].

To calculate a score for the Social Responsiveness Scale, a questionnaire was completed in 15 to 20 minutes. A total score of 76 or higher was considered severe and strongly associated with a clinical diagnosis of autistic disorder. A score of 60–75 was interpreted as falling in the mild to moderate range of social impairment [13].

### Sample collection

After an overnight fast, 10-ml blood samples, from both groups of children, were collected in test tubes containing sodium heparin as an anticoagulant. The tubes were centrifuged at 3500 rpm for 15 minutes at room temperature. Plasma and red blood cells were separated and stored at - 80°C until required for analysis.

### Biochemical analysis

Plasma is a complex bodily fluid containing proteins, peptides, lipids and metabolites and can reflect physiological activity and pathology in various body organs, including the CNS. In humans, approximately 500 ml of CSF is absorbed into the blood daily, making blood a suitable source of biomarkers of neurodegenerative or neurodevelopmental diseases [14]. All biochemical assays were performed in duplicate and blinded to the clinical status of the participants.

#### *1- Measurement of mercury*

The concentration of inorganic mercury (Hg) in red blood cells was determined by the method described by Magos [15] using a flameless atomic-absorption method. The red blood cells were diluted with saline to 20 ml, followed by the addition of 1 ml of a 1% cysteine solution, 10 ml of 8 M H_2_SO_4_ and 1 ml of SnCl_2_ (100 mg/ml). The sample was subjected to immediate aeration at a constant rate of 2.5 l/min through the reaction vessel, and 20 ml of a 45% NaOH was added. The SnCl_2_ reagent was used to release all of the inorganic mercury from the samples. Aeration was discontinued after the recorder pen had settled back to within a few chart divisions (2 or 3) of its original baseline, which was approximately 1 to 1.5 min, depending on the actual aeration rate. The concentration of mercury was measured using a flameless atomic-absorption method, and the concentration was calculated using a standard calibration curve prepared using a standard Hg concentration.

#### *2- Measurement of Pb*

Lead levels were measured in red blood cells using adaptations of the methods described by Miller et al. [16] and Parsons and Slavin [17]. Briefly, RBCs (0.1 ml) were resuspended and digested in 3.9 ml of 0.5 N nitric acid. Lead quantification was based on the measurement of light absorbed at 283.3 nm by the ground-state atoms of Pb from a hollow cathode lamp (HCl) source.

#### *3- Determination of glutathione-S-transferase activity (GST)*

GST activity was assessed in the plasma using an assay kit (Biovision, USA). The kit is based on a GST-catalyzed reaction between GSH and the GST substrate, CDNB (1-chloro-2, 4-dinitrobenzene). The GST-catalyzed formation of GS-DNB produces a dinitrophenyl thioether, which can be detected by a spectrophotometer at 340 nm. GST activity was expressed as μmole/min/ml plasma.

#### *4- Vitamin E assay (*α -tocopherol*)*

Plasma vitamin E was assessed using high pressure liquid chromatography (HPLC) as described by Driskell et al. [18]. The separation via HPLC follows an isocratic method at 30°C using a “reversed phase” column; one run lasts 15 minutes. The detection was performed with a UV detector at 290 nm. The quantification was performed with the delivered standard solution; the concentration was calculated via the integration of the peak areas in the internal standard calibration mode.

### Statistical analysis

The Statistical Package for the Social Sciences (SPSS) computer program was used. The results were expressed as the mean ± SD, and all statistical comparisons were made by means of independent t-tests, with *P* ≤0.05 considered as significant. ROC analysis was performed. Areas under the curve and cut off values were selected by the program. The degree of specificity and sensitivity were calculated. Moreover, the predictiveness diagrams of the four measured parameters were drawn using a Biostat 16 computer program in which the *x* axis represents percentile rank of the biomarker, the *y* axis represents the probability of identifying the disease and the horizontal line is the prevalence of the disease. Enter and stepwise multiple regression analyses were performed using CARS and SRS as two dependent variables and Hg, Pb, GST and vitamin E as independent variables.

## Results

**Figure 1:**
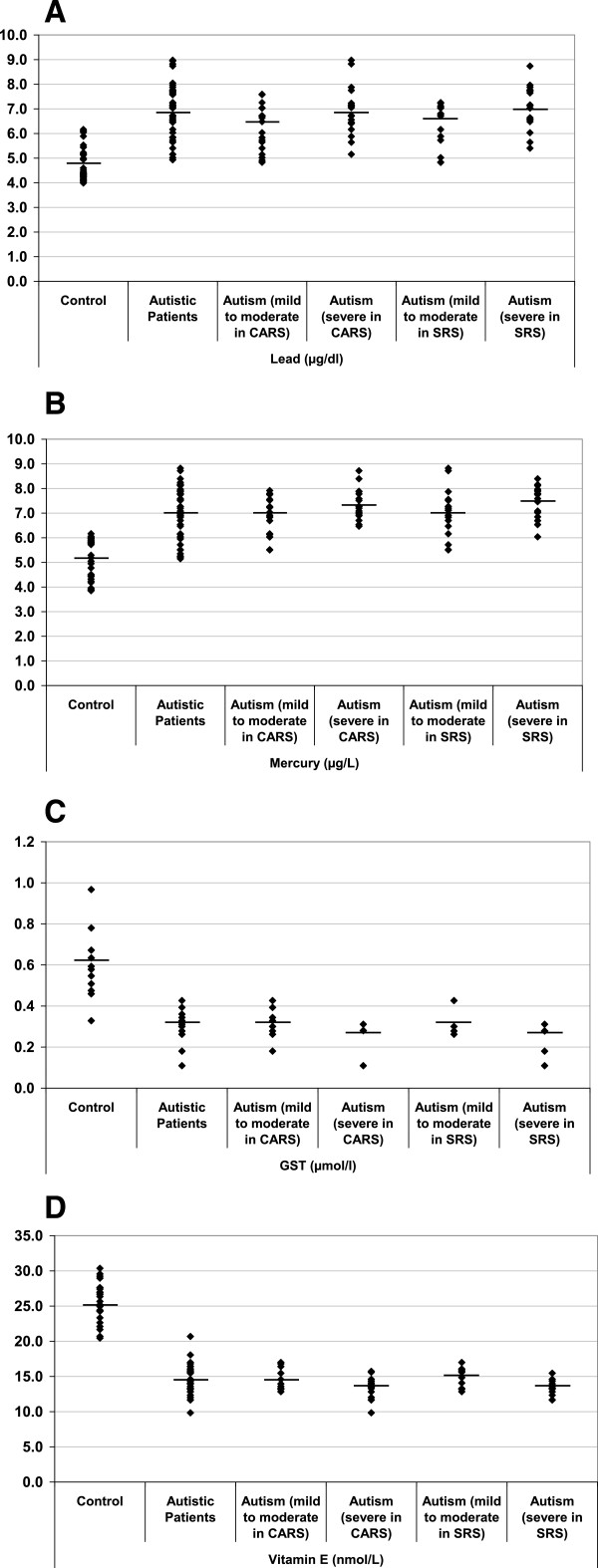
**Mean levels of A) mercury (μg/L), B) lead (μg/L), C) GST (μmol/min/ml) and D) vitamin E (nmol/L) of the autistic groups compared with age- and sex-matched controls.** The mean value for each group is designated by a line.

**Figure 2:**
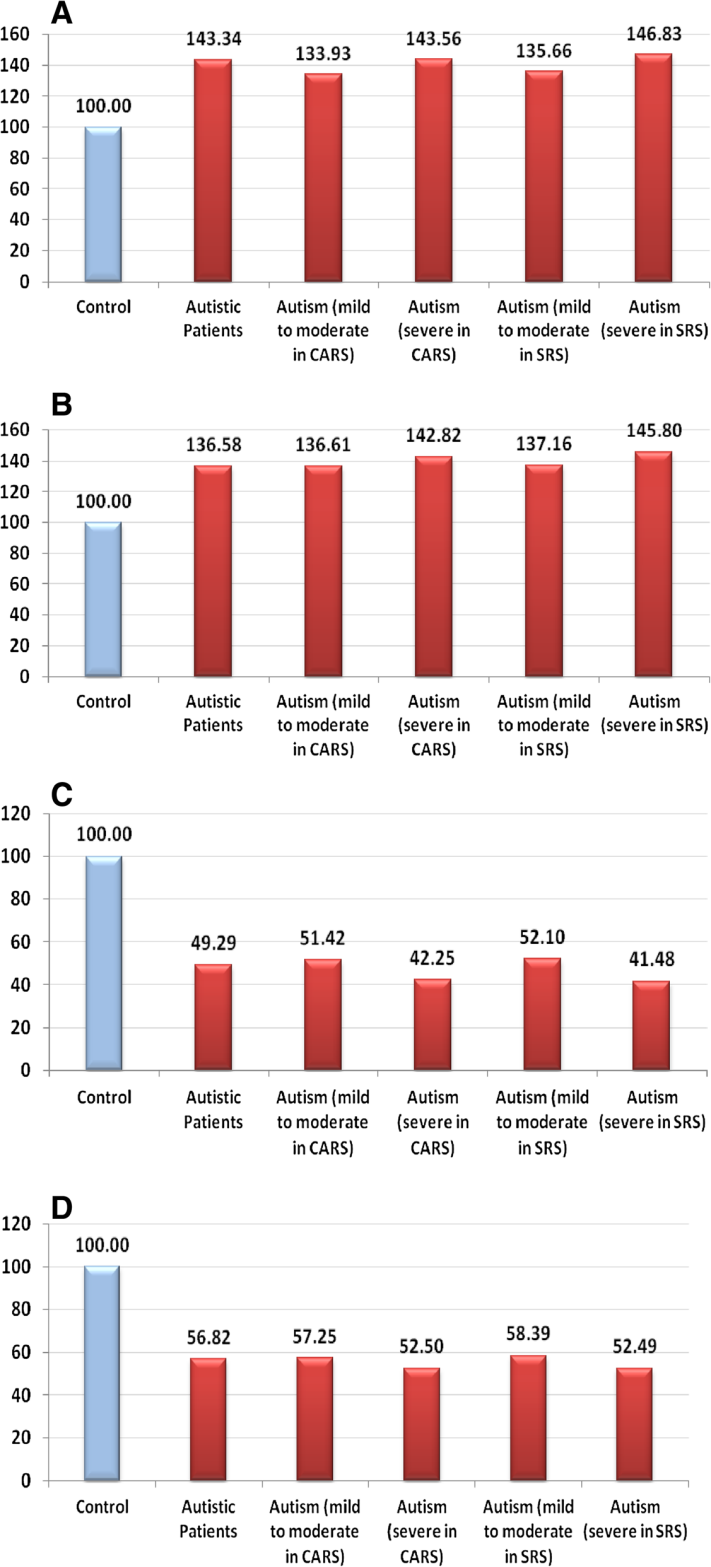
Percentage change of A) mercury, B) lead, C) GST and D) vitamin E in the autistic group relative to the control group, which is represented as 100%.

Table 1 and Figure 1 present the mean ± SD of Hg, Pb and plasma GST and vitamin E levels in RBCs of the control, severe autistic and mild-moderate autistic patients with different CARS and SRS scores. Mercury and Pb were significantly elevated in the autistic patients compared with the controls, with increases of 36.58% and 43.34%, respectively. GST and vitamin E levels decreased by 50.71% and 43.18% percent, respectively (Figure 2).

**Table 1:** Mercury (μg/L), lead (μg/dl), GST (μmol/l) and vitamin E (nmol/L) levels in the control & autistic groups

**Parameters**	**Group**	**N**	**Mean ± S.D.**	***P *** **value**^**a**^	***P *** **value**^**b**^
**Mercury (μg/L)**	Control	32	5.12 ± 0.83		
	Patients with autism	58	6.99 ± 0.94		0.001
	Autism (mild to moderate in CARS)	29	6.49 ± 0.65	0.042	0.001
	Autism (severe in CARS)	28	7.31 ± 0.49		0.001
	Autism (mild to moderate in SRS)	22	7.02 ± 0.85	0.050	0.001
	Autism (severe in SRS)	22	7.46 ± 0.59		0.001
**Lead (μg/dl)**	Control	32	4.73 ± 0.67		
	Patients with autism	58	6.79 ± 0.97		0.001
	Autism (mild to moderate in CARS)	29	6.34 ± 0.82	0.041	0.001
	Autism (severe in CARS)	28	6.80 ± 0.83		0.001
	Autism (mild to moderate in SRS)	22	6.42 ± 0.84	0.040	0.001
	Autism (severe in SRS)	22	6.95 ± 0.81		0.001
**GST (μmol/min/ml)**	Control	30	0.61 ± 0.17		
	Patients with autism	30	0.30 ± 0.08		0.001
	Autism (mild to moderate in CARS)	18	0.31 ± 0.07	0.043	0.001
	Autism (severe in CARS)	12	0.26 ± 0.07		0.001
	Autism (mild to moderate in SRS)	8	0.32 ± 0.07	0.027	0.001
	Autism (severe in SRS)	18	0.25 ± 0.06		0.001
**Vitamin E (nmol/L)**	Control	27	25.44 ± 2.62		
	Patients with autism	28	14.45 ± 2.28		0.001
	Autism (mild to moderate in CARS)	11	14.56 ± 1.58	0.048	0.001
	Autism (severe in CARS)	17	13.36 ± 1.47		0.001
	Autism (mild to moderate in SRS)	11	14.85 ± 1.36	0.044	0.001
	Autism (severe in SRS)	12	13.35 ± 1.14		0.001

^a^*P* value between mild to moderate and severe in CARS and SRS.

^b^*P* value between control and autistic groups.

**Figure 3:**
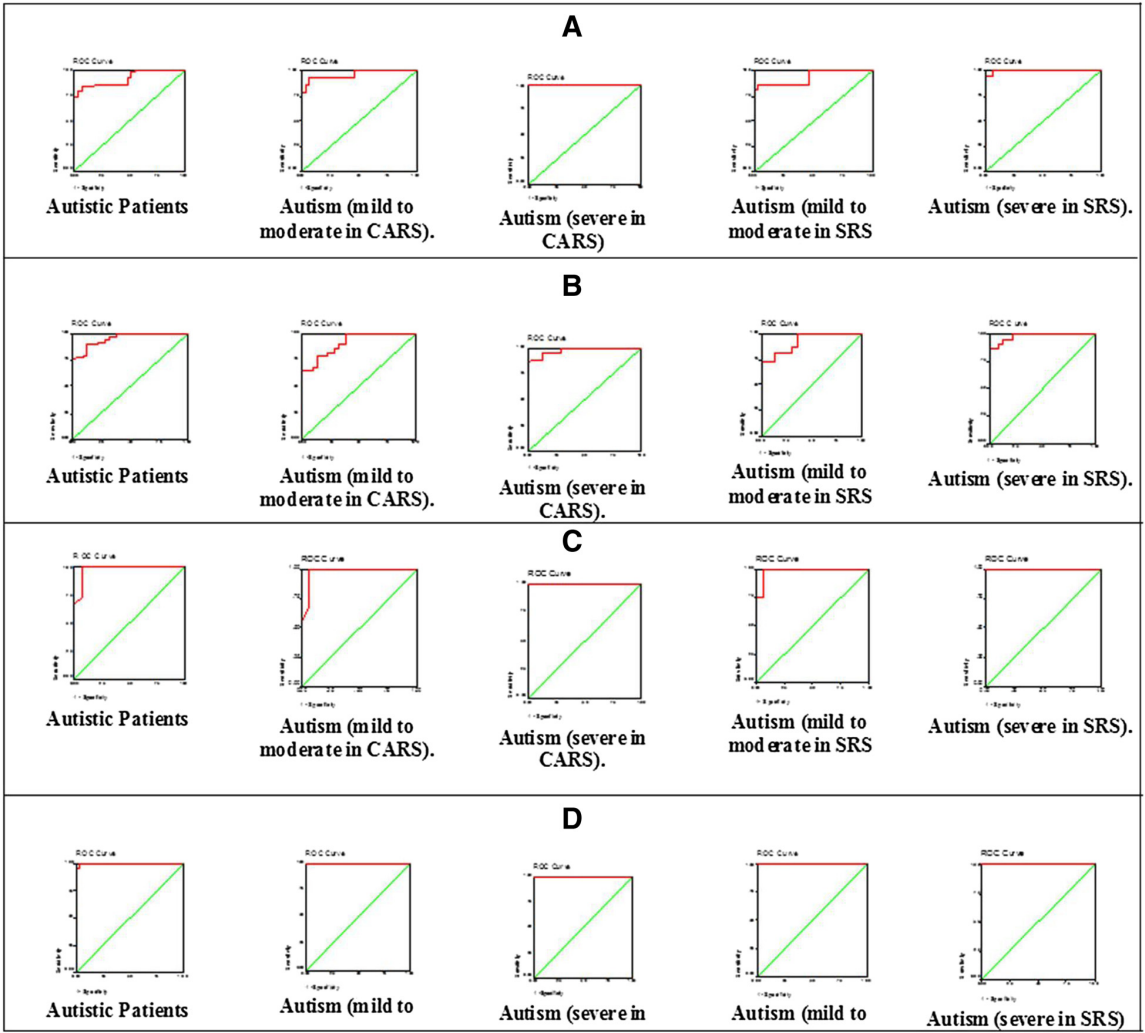
ROC-curves for A) mercury, B) lead, C) GST and D) vitamin E in the autistic groups.

**Figure 4:**
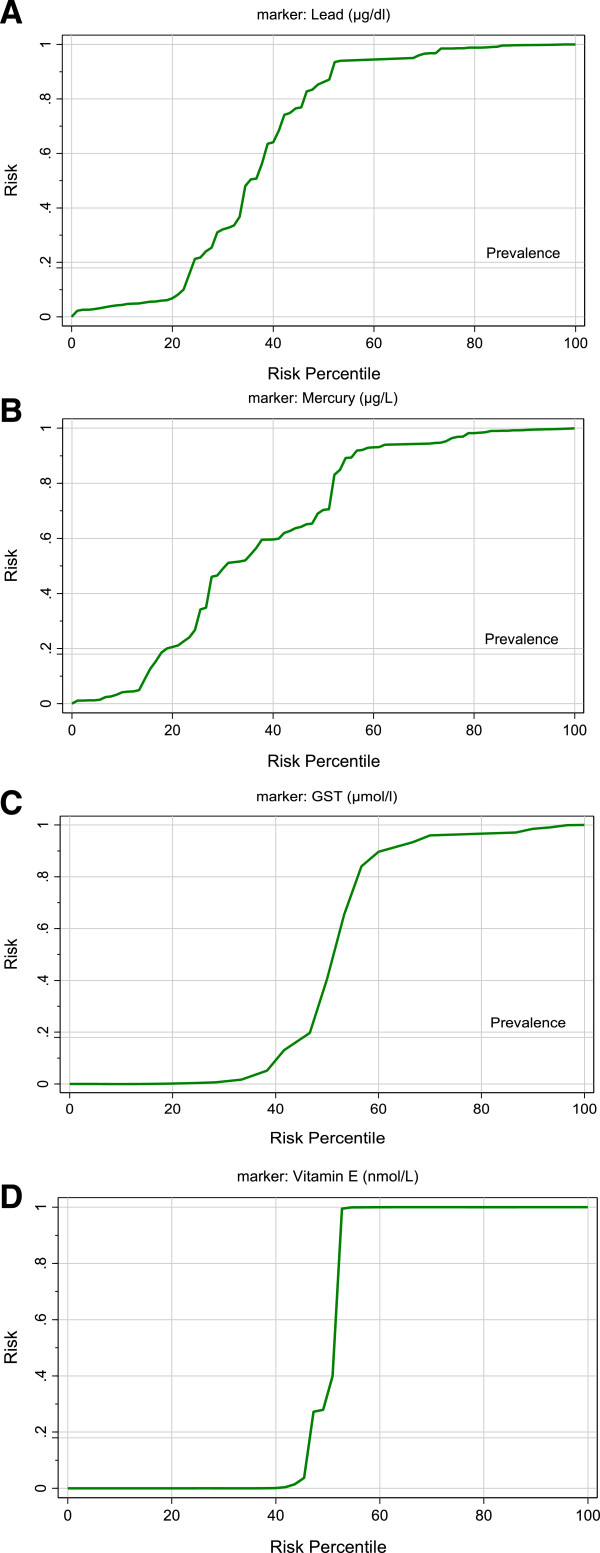
Predictiveness curve for A) mercury, B) lead, C) GST and D) vitamin E in the autistic patients.

Table 2 and Figure 3 show the ROC analysis of the four measured parameters. The area under the curve (AUC), specificity and sensitivity are illustrated. Figure 4 shows the predictiveness curves of the four measured parameters exhibiting high and low risk in relation to the prevalence of autism in Saudi Arabia.

**Table 2:** ROC-curve of mercury (μg/L), lead (μg/dl), GST (μmol/l) and vitamin E (nmol/L) levels in the autistic groups

**Parameters**		**Autistic patients**	**CARS**		**SRS**	
			**Mild to moderate**	**Severe**	**Mild to moderate**	**Severe**
**Mercury**	Area under the curve	0.926	0.961	1.000	0.935	0.997
	Best cutoff value	6.018	6.018	6.316	6.105	6.346
	Sensitivity %	84.5%	93.1%	100.0%	86.4%	95.5%
	Specificity %	93.8%	93.8%	100.0%	96.9%	100.0%
**Lead**	Area under the curve	0.953	0.917	0.980	0.923	0.980
	Best cutoff value	5.586	5.590	6.132	6.132	6.321
	Sensitivity %	89.7%	79.3%	89.3%	72.7%	86.4%
	Specificity %	87.5%	87.5%	96.9%	96.9%	100.0%
**GST**	Area under the curve	0.980	0.974	1.000	0.983	1.000
	Best cutoff value	0.443	0.443	0.320	0.443	0.320
	Sensitivity %	100.0%	100.0%	100.0%	100.0%	100.0%
	Specificity %	93.3%	93.3%	100.0%	93.3%	100.0%
**Vitamin E**	Area under the curve	0.999	1.000	1.000	1.000	1.000
	Best cutoff value	19.254	18.718	18.089	18.718	17.953
	Sensitivity %	96.4%	100.0%	100.0%	100.0%	100.0%
	Specificity %	100.0%	100.0%	100.0%	100.0%	100.0%

Table 3 shows positive and negative correlations between the four measured parameters in this study. The results showed that while Pb and Hg were positively correlated with each other, both metals were negatively correlated with GST and vitamin E as enzymatic and non-enzymatic detoxifiers.

**Table 3:** Statistically significant Pearson’s correlation between the four measured parameters

**Parameters**	**R (Person correlation)**	**Sig.**	
GST ~ Mercury	-0.613	0.001^**^	N^b^
GST ~ Lead	-0.588	0.001^**^	N^b^
GST ~ Vitamin E	0.696	0.001^**^	P^a^
Mercury ~ Lead	0.761	0.001^**^	P^a^
Mercury ~ Vitamin E	-0.710	0.001^**^	N^b^
Lead ~ Vitamin E	-0.715	0.001^**^	N^b^

^**a**^Positive correlation.

^**b**^Negative correlation.

^**^Correlation is significant at the 0.01 level (2-tailed).

Tables 4, 5, 6 and 7 show multiple regression analysis results using the Enter method (Tables 4 & 6) and Stepwise method (Tables 5 & 7) for the CARS and SRS scores as two dependent variables, respectively. The value of R^2^ shows that 0.56 or 56% of the variance of the CARS scores and almost 40% of the variance of the SRS scores were explained by the regression of the four measured parameters, with GST and vitamin E being more predictive (stepwise regression as presented in Tables 5 & 7).

**Table 4:** Multiple regression using the enter method for CARS as a dependent variable

**Model**	**Beta**	**P value**	**Adjusted R**^**2**^	**Model**	
				**F value**	**P value**
(Constant)	3.038	0.005	0.564	25.907	0.001
Mercury (μg/L)	0.161	0.139			
Lead (μg/dl)	0.004	0.971			
GST (μmol/l)	-1.159	0.081			
Vitamin E (nmol/L)	-0.082	0.002			

**Table 5:** Multiple regression using the stepwise method for CARS as a dependent variable

**Model**	**Beta**	**P value**	**Adjusted R**^**2**^	**Model**	
				**F value**	**P value**
(Constant)	4.517	0.001	0.560	49.909	0.001
Vitamin E (nmol/L)	-0.103	0.001			
GST (μmol/l)	-1.383	0.035			

**Table 6:** Multiple regression using the enter method for SRS as a dependent variable

**Model**	**Beta**	**P value**	**Adjusted R**^**2**^	**Model**	
				**F value**	**P value**
(Constant)	4.844	0.028	0.391	12.867	0.001
Mercury (μg/L)	0.261	0.248			
Lead (μg/dl)	0.117	0.575			
GST (μmol/l)	-1.890	0.185			
Vitamin E (nmol/L)	-0.134	0.012			

**Table 7:** Multiple regression using the stepwise method for SRS as a dependent variable

**Model**	**Beta**	**P value**	**Adjusted R**^**2**^	**Model**	
				**F value**	**P value**
(Constant)	6.298	0.001	0.384	47.167	0.001
Vitamin E (nmol/L)	-0.202	0.001			

## Discussion

Exposure to even low levels of lead (Pb) early in life has adverse effects on a variety of cognitive and behavioral functions and neurochemical systems, resulting in deficits in learning, memory and attention that may persist into adulthood [19-22]. Persistent effects of Pb exposure early in life can produce changes that arise from physiological re-programming [23]. In this study, there was a significant increase in Pb levels in patients with ASD compared with the control group, coupled with a correlation between Pb concentration and the severity of SRS and CARS scores. These observations support a recent study on patients with ASD by Schneider et al. (2013) [24], who suggested potential epigenetic effects of developmental Pb exposure on DNA methylation. These effects were mediated at least partially through the dysregulation of methyltransferases as multiple forms of proteins, at least some of which are potentially involved in cognition [25], and the resulting abnormalities were recorded in patients with ASD [26].

The reported elevation of Hg and Pb in the RBCs of patients with ASD compared with the control subjects (Table 1 and Figure 1A&B) can be related to and support a previous study by Al-Yafee et al. [27] in which Saudi patients with ASD were described as poor detoxifiers with a lower GSH/GSSG ratio and remarkably less active GST and thioredoxin reductase as markers of the detoxification mechanism. It is well known that GSH and GST are both critical for the detoxification of mercury [28]. While GSH carries Hg through biliary transport for excretion, Hg^2+^ rapidly oxidizes glutathione [28]. This observation is correlated with the previous work of Al-Gadani et al. [8] who reported lower GSH in the plasma of Saudi patients with ASD. Additionally, the increase of Hg and Pb recorded in this study is consistent with previous studies. For example, Blaurock-Busch et al. [29] found a significant increase of both toxicants in the hair of autistic children compared with non-autistic children. This observation may indicate an impaired detoxification mechanism as a risk factor that significantly contributes to the etiology of autism. The exposure of patients with ASD in early childhood to mercury vapor, methylmercury and ethylmercury may occur through dental amalgams, fish intake and vaccinations [30-32].

Furthermore, in this study, remarkably higher levels of Hg and Pb were recorded in patients with severe social and cognition impairment (SRS & CARS) compared with those with mild-moderate abnormalities. This observation suggests that heavy metal toxicity is closely related to the pathophysiology of autism. This outcome does not concur with a study by Elsheshtawy et al. in which Hg, but not Pb, was found in the hair of autistic patients as a biochemical correlate to disease severity (CARS) [33]. However, our results are consistent with the recent work of Adams et al. [34] who reported that children with autism have higher average levels of several toxic metals, among which Hg and Pb are strongly associated with variations in the severity of the disorder. However, in their study, Adams et al. found a non-significant difference of Hg in children with autism vs. neurotypical children. This could be attributed to differences in geographic exposure to mercury (Saudi Arabia vs. Arizona). Patients with autism are poor detoxifiers (i.e., unable to detoxify mercury when it reaches a certain level) [27]. Therefore, this could indicate a higher rate of exposure to Hg in the Saudi population compared with the population in Arizona.

Face recognition is a core deficit of social impairment in autism. A number of studies indicate that norepinephrine (NE) and dopamine (DA) modulate and reduce behavioral responses to changes in the social environment [35,36]. In addition, serotonin (5HT) transporter binding appears to be reduced in certain brain regions known to play an important role in social cognition and behavior [37], and 5HT binding potential is negatively correlated with social impairment. Therefore, recording Pb as a correlate to severity of SRS and CARS scores in the present study is consistent with the recent findings of El-Ansary et al. [38] in which a positive association was observed between chronic Pb toxicity and lower levels of neurotransmitters as markers of neurologic injury in autistic brains in a Saudi population. Alternately, the biochemical correlation between Pb and the severity of autism is in agreement with other studies. Szkup-Jabłońska et al. [39] and Blaurock-Busch et al. [40] reported a significant correlation between Pb and fear, nervousness, verbal and nonverbal communication, social activity level, and consistency of intellectual response. Moreover, the positive correlation between elevated Pb toxicity and both autistic scales could be supported by the fact that Pb exposure affects multiple health outcomes and physiological systems [41]. These include behavioral/cognitive/IQ effects, nerve conductive effects, hearing loss, reproduction and development effects and death from encephalopathy. Long-term trends in population exposure to Pb (indexed through use of leaded petrol and paint) were remarkably consistent with the link between IQ and social behavior [42].

The significant reduction in plasma GST in Saudi patients with autism compared with controls is documented in this study (Table 1 and Figure 1C&D). This could be related to the significant depletion of GSH as a substrate of GST in the plasma of patients with ASD compared with control subjects [8,43,44]. The reduction in this essential detoxifying enzyme can explain the poor detoxification in patients with ASD, leading to the Hg and Pb toxicity discussed above.

In our study, there was an inverse relationship between decreased levels of plasma GST and the severity of autism, as measured by the SRS and CARS. Severely autistic cases had a remarkably lower GST activity compared to mild-moderate cases of autism. These outcomes concur with Geier et al. [45], who also found a significant inverse relationship between blood GSH levels and autism severity measured with the CARS. Mercury aggravates impaired glutathione synthesis by depleting glutathione in lymphocytes and monocytes, leading to an increased risk of immuno and cytotoxic effects.

Although, the roles and importance of various forms of vitamin E are still unclear [46], it has been suggested that the most important function of α-tocopherol is as a signaling molecule playing an important role in protecting neurons from damage [47]. As an antioxidant, vitamin E may prevent or reduce the propagation of free radicals, which are associated with physical decline, in the human body. This may help reduce muscle or DNA damage and prevent the development of pathological conditions, such as autism [48]. Table 1 and Figure 1D demonstrate the significantly reduced levels of vitamin E in the plasma of patients with ASD compared with the control group. Herndon et al. [49] also found decreased vitamin E levels in autistic patients. The brain contains high levels of oxidizable lipids that must be protected by antioxidants; hence, the supplementation of ASD patients with vitamin E as a major lipophilic antioxidant could be helpful [7,50]. The highly significant correlation between vitamin E depletion and severity of autism, as measured by the SRS and CARS, supports its critical role in protecting against the toxic effects of Pb and Hg. This is consistent with a previous report by Adams et al. [7] that showed a significant association between vitamin E insufficiency and the severity of the Autism Scale (SAS).

ROC analyses of Pb, Hg, GST and vitamin E are presented in Table 2 and Figure 3(A-D). All measured parameters demonstrated almost 100% sensitivity and very high specificity, which also confirmed the hypothesis that autistic patients are poor detoxifiers, unable to readily excrete toxic substances (e.g., Hg and Pb), and suggests that reduced GST activity and depleted vitamin E are two critical factors related to poor detoxification.

Lead, Hg, GST and vitamin E show perfect predictiveness curves (Figure 4A-D). Excellent predictiveness curves for the four parameters reflect the possibility of using any of these parameters to follow up an antioxidant-related treatment strategy. A successful treatment could be followed through a remarkable elevation of plasma vitamin E, the activation of GST or both in autistic patients. Alternately, efficacious treatment could occur through a reduction in Pb and Hg levels. In addition, the relationship between vitamin E deficiency and the etiology of autism could be ascertained by the high specificity, sensitivity and AUC, as shown with the ROC analysis (Table 2).

The negative correlations between Hg & Pb and vitamin E & GST (Table 3) suggest the use of vitamin E as a non-enzymatic antioxidant in treating patients with autism. This suggestion is supported by the multiple regression analysis results (Tables 4, 5, 6 and 7), confirming that higher levels of Hg and Pb, together with lower levels of GST and vitamin E, can be used to predict cognitive and social impairment with the regression of both antioxidant parameters, which is more related to abnormalities of both.

## Conclusion

The high values of both sensitivity and specificity recorded for Pb, Hg, GST and vitamin E, together with the good predictiveness curves; suggest that these can be used as biomarkers for measuring the severity of SRS and CARS scores in a Saudi population. This study confirmed the impaired antioxidant and detoxification mechanisms in Saudi autistic patients. Hence, early intervention through the supplementation of good quality and safe antioxidants, including vitamin E, carnosine and selenium, can be helpful in decreasing the burden of heavy metal toxicity. Vitamin E exists in eight different forms: four tocopherols and four tocotrienols. The measured form of vitamin E, α –tocopherol, is one of the forms that regulate signal transduction pathways by mechanisms that are independent of its antioxidant properties, and its use as a supplement can be effective in reducing the toxicity burden in these patients. Autistic children who undergo intensive intervention have better social interaction than children who do not.

## Competing interests

The author declares that they have no competing interest.

## Authors’ contributions

AA performed the practical work and co-drafted the manuscript. LA provided samples and participated in the diagnosis of the autistic samples. AE designed the study and drafted the manuscript. All authors have read and approved the final manuscript.
